# Preliminary Genotoxicity Assessment of Calcium Phosphate Cement Incorporated with Palm Tocotrienol Using Bacterial Reverse Mutation Assay

**DOI:** 10.3390/biomedicines14051095

**Published:** 2026-05-12

**Authors:** Sok Kuan Wong, Siti Sarah Md Dali, Kok-Yong Chin, Fairus Ahmad, Abdul Hadi Ariffin, Farah Md Fauzi, Muhd. Zulkarnain Mahmud, Ilyana Hakimi Ahmad Sabri

**Affiliations:** 1 Department of Pharmacology, Faculty of Medicine, Universiti Kebangsaan Malaysia, Jalan Yaacob Latif, Bandar Tun Razak, Cheras, Kuala Lumpur 56000, Malaysia; p117856@siswa.ukm.edu.my (S.S.M.D.); chinky@ukm.edu.my (K.-Y.C.); 2 Department of Anatomy, Faculty of Medicine, Universiti Kebangsaan Malaysia, Jalan Yaacob Latif, Bandar Tun Razak, Cheras, Kuala Lumpur 56000, Malaysia; fairusahmad@ukm.edu.my (F.A.); ilyana@nibm.my (I.H.A.S.); 3 Malaysian Institute of Pharmaceuticals & Nutraceuticals, National Institutes of Biotechnology Malaysia, Halaman Bukit Gambir, Gelugor, Penang 11700, Malaysia; hadi@nibm.my (A.H.A.); farah@nibm.my (F.M.F.); zulkarnain@nibm.my (M.Z.M.)

**Keywords:** Ames test, biomaterials, bone regeneration, *Elaeis guineensis*, vitamin E

## Abstract

**Background/Objectives**: Calcium phosphate cement (CPC) is extensively utilised in bone repair owing to its biocompatibility, osteoconductivity, and compositional similarity to native bone. Functionalisation of CPC with palm tocotrienol may enhance its regenerative potential. However, the incorporation of phytochemicals requires safety evaluation to exclude potential genotoxic risks. This study investigated the mutagenic potential of CPC and tocotrienol-enriched CPC (CPC-T3) using the bacterial reverse mutation assay. **Methods**: Mutagenicity was evaluated in five bacterial strains, including *Salmonella typhimurium* TA100, TA98, TA1535, TA1537, and *Escherichia coli* WP2 *trp uvrA*, under both non-metabolic and metabolic activation conditions. Revertant colonies were quantified at multiple concentrations and mutagenicity ratios were calculated relative to the negative control. **Results**: Across all strains and metabolic conditions, neither CPC nor CPC-T3 induced reproducible or concentration-dependent increases in revertant colony numbers. Although isolated elevations were detected at certain concentrations, these findings lacked dose–response relationships and did not meet the criteria for a positive mutagenic response according to Organisation for Economic Co-operation and Development (OECD) Test Guideline No. 471. The performance of negative and positive controls confirmed the validity and sensitivity of the assay. Notably, the inclusion of palm tocotrienol did not alter the overall mutagenicity profile of CPC. **Conclusions**: CPC and CPC-T3 demonstrated no evidence of mutagenic activity under the conditions of the bacterial reverse mutation assay. These findings represent preliminary genotoxicity screening. Further mammalian genotoxicity and in vivo studies are warranted to support future translational development as implantable medical devices.

## 1. Introduction

Bone defects resulting from trauma, tumour resections, congenital abnormalities, infections and degenerative diseases remain a major clinical challenge in orthopaedic and dental practice. Although bone tissue possesses intrinsic regenerative capacity for small injuries, extensive or critical-sized defects typically exceed this self-repair potential and necessitate the application of bone grafts or implantable materials to establish structural stability and functional recovery [1]. Autologous bone grafting is considered the preferred therapeutic approach for bone defect reconstruction. Nevertheless, its clinical application is constrained by the limited quantity of harvestable bone and the risk of morbidity and postoperative complications at both the donor and implantation sites [2]. Hence, synthetic bone substitute materials have attracted increasing attention as alternative strategies for bone repair and regeneration.

Calcium phosphate cement (CPC) has been extensively employed in bone repair applications due to its excellent biocompatibility, osteoconductivity, and chemical similarity to the inorganic mineral phase of native bone tissue [3]. CPC is a paste formed by combining a powder phase with a liquid component, resulting in a mouldable and injectable material capable of filling irregular bone defects. It undergoes in situ setting and hardening, making it particularly suitable for minimally invasive surgical procedures [4]. Despite these favourable characteristics, conventional CPC formulations often possess limited intrinsic biological activity and may require further functionalisation to enhance their regenerative potential. Incorporation of bioactive compounds into CPC represents a promising approach to improve bone healing outcomes.

Tocotrienols, members of the vitamin E family isolated from the oil palm (*Elaeis guineensis*), are widely recognised for their strong antioxidative, anti-inflammatory, and osteoprotective properties [5,6]. Previous studies have demonstrated that tocotrienols promote bone formation through modulation of key regulatory pathways, including the receptor activator of nuclear factor kappa-B (RANK)/receptor activator of nuclear factor kappa-B ligand (RANKL)/osteoprotegerin (OPG) axis, the canonical Wnt/β-catenin signalling pathway, and oxidative stress-related mechanisms [7]. These mechanistic insights underscore their potential value as bioactive additives in bone-regenerative biomaterials. Nevertheless, the incorporation of phytochemical compounds into implantable materials necessitates rigorous safety assessment, as degradation products or metabolically activated intermediates may pose potential genotoxic risks.

Genetic toxicology assessment is an essential component in the preclinical evaluation of biomaterials intended for clinical application. The bacterial reverse mutation assay, also known as the Ames test, is a widely recognised method for evaluating mutagenic activity arising from chemical substances or bioactive additives. This assay employs multiple strains of *Salmonella typhimurium* and *Escherichia coli* that are genetically modified to require specific amino acids for growth. Testing is conducted in the presence and absence of metabolic activation to identify substances capable of inducing gene mutations that restore the bacteria’s ability to synthesise essential amino acids [8].

To date, information regarding the mutagenic safety profile of CPC formulations incorporating palm tocotrienol is limited. Establishing the genetic safety of biofunctionalised cement systems is crucial prior to further translational and clinical development. This present study aimed to conduct an initial in vitro assessment of the mutagenic potential of CPC with and without palm tocotrienol incorporation using the bacterial reverse mutation (Ames) assay. In vitro assays are widely used as early-stage screening tools due to their sensitivity, reproducibility, and ethical advantages, and they form part of a tiered approach to biological evaluation. The assessment was conducted in five bacterial strains (*S. typhimurium* TA100, TA98, TA1535, TA1537, and *E. coli* WP2 *trp uvrA*) under both non-activated and metabolic-activated conditions. The findings of this study provide important preclinical evidence supporting the genetic safety of tocotrienol-enhanced CPC for potential bone regenerative applications.

## 2. Materials and Methods

### 2.1. Preparation of CPC

Monocalcium phosphate monohydrate (MCPM) (Sigma Aldrich, Darmstadt, Germany), beta-tricalcium phosphate (β-TCP) (Sigma Aldrich, Germany), citric acid (Sigma Aldrich, Germany), and sodium hyaluronate (BBI Life Sciences, Shanghai, China) were subjected to sterilisation using gamma (γ) radiation at a dose of 25 kGy prior to use. To formulate the CPC, MCPM and β-TCP were mixed at a mass ratio of 45:55. Citric acid was subsequently incorporated into the powder mixture with a weight ratio of 1:80 to improve the setting characteristics of the cement. The CPC used in this study was prepared from commercially available/research-grade raw materials with established composition and quality, as reported by the supplier and supported by previous studies [9,10].

The resulting powder mixture was combined with a 2% (*w*/*v*) sodium hyaluronate solution prepared in distilled water to produce a homogeneous paste. This paste was further mixed with an oil-based suspension, either containing palm tocotrienol or without supplementation, at an oil-to-powder ratio of 0.35 g/g. The formulations containing tocotrienol and those without supplementation were designated as CPC-T3 and CPC, respectively.

Palm tocotrienol, isolated from *Elaeis guineensis*, (batch number: 19060070A0) was a kind gift from Davos Life Science Sdn. Bhd. (Port Klang, Selangor, Malaysia). The composition of the tocotrienol fraction consisted of 28.3% α-tocopherol, 26.8% α-tocotrienol, 2.3% β-tocotrienol, 29.5% γ-tocotrienol, 8.3% δ-tocotrienol, and 4.6% α-tocomonoenol.

### 2.2. Preparation of Test Item Extracts

The CPC formulations with and without palm tocotrienol were extracted using nutrient broth at a ratio of 0.2 g/mL in a sterile container for 24 h at 37 °C. These conditions are commonly used to simulate the release of leachable substances under physiological conditions. Upon completion of extraction, the solutions were sterilised by filtration through a 0.2 μm membrane filter to remove particulate matter and microbial contaminants. The resulting filtrates were designated as 100% stock concentration. Serial dilutions of the sterile extracts were prepared using nutrient broth to obtain final concentrations of 50%, 25%, 12.5%, and 6.75%, allowing assessment of potential dose-dependent effects across a range of exposure. This approach reflects standard practice in biomaterial safety assessment, where exposure is defined by extract concentration rather than individual component quantification. All test concentrations were used immediately for the bacterial reverse mutation assay.

### 2.3. Bacterial Strains and Culture Preparation

The bacterial strains employed in this study included *S. typhimurium* TA100 (Molecular Toxicology Inc., Boone, NC, USA), *S. typhimurium* TA98 (Molecular Toxicology Inc., USA), *S. typhimurium* TA1535 (American Type Culture Collection, Manassas, VA, USA), and *S. typhimurium* TA1537 (American Type Culture Collection, USA), and *E. coli* WP2 *trp uvrA* (Molecular Toxicology Inc., USA). *S. typhimurium* TA100, TA1535 and *E. coli* WP2 *trp uvrA* are used to detect mutagens that lead to base-pair substitutions whereas *S. typhimurium* TA98 and TA1537 are used to identify genotoxins that induce frameshift mutations (Table 1). Bacterial stock cultures (100 μL) were inoculated into 10 mL of nutrient broth and incubated at 4 °C for four hours to allow for gradual revival. The cultures were subsequently incubated in a shaking incubator at 37 °C for 16 h to achieve the appropriate cell density for the assay.

### 2.4. Control Treatment

Sterile distilled water and dimethyl sulfoxide (DMSO) were used as negative controls for aqueous and solvent-based test samples, respectively. Positive controls were selected based on the mutation type, including sodium azide (Sigma Aldrich, Germany), 9-aminoacridine (Sigma Aldrich, Germany), 2-nitrofluorene (Sigma Aldrich, Germany), and methyl methanesulfonate (Sigma Aldrich, Germany) for assays without metabolic activation, as well as 2-aminoanthracene (Sigma Aldrich, Germany) for assays with metabolic activation.

### 2.5. Preparation of Metabolic Activation System

The liver microsomal metabolic activation (S9) fraction was obtained from liver homogenates of Sprague-Dawley rats. It was employed in assays requiring metabolic activation to simulate mammalian liver metabolism. The S9 mixture was freshly prepared prior to use and contained necessary cofactors for metabolic activation, including nicotinamide adenine dinucleotide phosphate (NADP), glucose-6-phosphate, magnesium chloride (MgCl_2_), and potassium chloride (KCl). The final concentration of S9 in the assay was 5% (*v*/*v*).

### 2.6. Bacterial Reverse Mutation Assay (Ames Test)

A total of 100 μL of each test item extract was combined with 500 μL of phosphate buffer (for assays without metabolic activation) or S9 mixture (for assays with metabolic activation). Subsequently, 100 μL of the corresponding bacterial strain suspension was added. Each concentration, along with the positive and negative controls, was tested in triplicate.

The reaction mixtures were incubated at 37 °C with gentle shaking for 30 min. Following pre-incubation, 2 mL of molten top agar (maintained at 45 °C) was added to each tube. The entire mixture was immediately poured onto pre-warmed minimal glucose agar plates and gently rotated to ensure uniform distribution. The plates were then incubated at 37 °C for 72 h. After incubation, the number of revertant colonies on each plate was counted using an automated colony counter. The data were recorded to evaluate mutagenic potential in accordance with Organisation for Economic Co-operation and Development (OECD) Test Guideline No. 471. Mutagenic ratio was calculated based on fold increases in revertant colony numbers of test item relative to the negative control (1).

(1) $$Mutagenicity\;Ratio=\frac{Mean\;revertant\;colonies\;(test\;item)}{Mean\;revertant\;colonies\;(negative\;control)}$$

### 2.7. Analysis

Data were expressed as mean ± standard deviation for each test concentration based on three independent replicates. The normality of data distribution was assessed using Shapiro–Wilk test. Differences between each test concentration and the corresponding negative control were analysed separately for each bacterial strain and metabolic activation condition. Comparisons between CPC and CPC-T3 were also performed. Statistical analysis was conducted using mixed-design analysis of variance (ANOVA) with small effect analysis. A *p*-value < 0.05 was considered statistically significant. Inhibitory or toxic effects on bacterial growth was determined by a reduction in the number of revertant colonies relative to the negative control. Mutagenicity was considered positive when the mutagenicity ratio was ≥2, and a clear dose–response relationship was observed.

## 3. Results

The mutagenic potential of CPC with and without palm tocotrienol incorporation was assessed using the bacterial reverse mutation assay across five bacterial strains (including *S. typhimurium* TA100, TA98, TA1535, TA1537, and *E. coli* WP2 *trp uvrA*) under both non-activated and metabolic-activated conditions.

**Figure 1:**
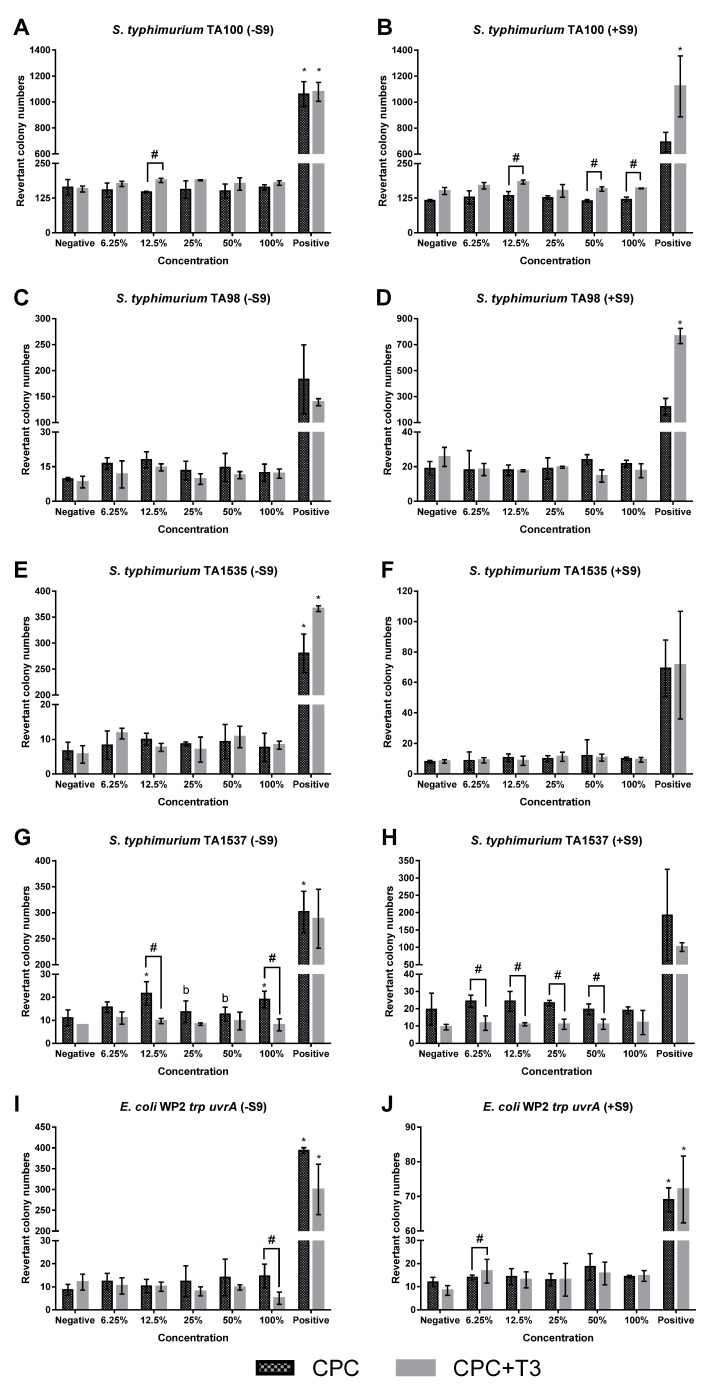
Revertant colonies of bacteria induced by CPC and CPC-T3 in the absence and presence of metabolic activation. (**A**). *S. typhimurium* TA100 (without metabolic activation), (**B**). *S. typhimurium* TA100 (with metabolic activation), (**C**). *S. typhimurium* TA98 (without metabolic activation), (**D**). *S. typhimurium* TA98 (with metabolic activation), (**E**). *S. typhimurium* TA1535 (without metabolic activation), (**F**). *S. typhimurium* TA1535 (with metabolic activation), (**G**). *S. typhimurium* TA1537 (without metabolic activation), (**H**). *S. typhimurium* TA1537 (with metabolic activation), (**I**). *E. coli* WP2 *trp uvrA* (without metabolic activation), and (**J**). *E. coli* WP2 *trp uvrA* (with metabolic activation). Data are presented as mean ± standard deviation. Symbol ‘*’ indicates significant difference as compared to the negative control; ‘#’ indicates significant difference as compared to CPC at the same concentration; ‘b’ indicates significant difference as compared to 12.5%.

Across all strains, the revertant colony counts in negative control groups were consistent with ranges reported in the literature for the respective tester strains. The positive controls produced marked increases in revertant colonies under both metabolic conditions, confirming the sensitivity and validity of the assay. Exposure to CPC and CPC-T3 at concentrations ranging from 6.75% to 100% did not result in statistically significant increases in revertant colony counts compared with the negative control in all bacterial strains, regardless of metabolic activation status (Figure 1).

In *S. typhimurium* TA100, mutagenicity ratios remained below 1.23 across all tested concentrations under both non-activated and activated conditions, with no evidence of a dose-dependent trend (Table 2a). In *S. typhimurium* TA98, mutagenicity ratios ranged from 1.18 to 1.86 in the absence of metabolic activation, whereas values remained close to unity under activated conditions (Table 2b). In *S. typhimurium* TA1535, mutagenicity ratios exceeding 2.0 were observed at 6.25% and 50% CPC-T3 under non-activated conditions. However, this response was isolated, lacked dose dependency, and was not reproduced at higher concentrations. Under metabolic activation, mutagenicity ratios remained below 1.34 across all concentrations (Table 2c).

In *S. typhimurium* TA1537, a mutagenicity ratio exceeding 2.0 was noted at 12.5% CPC without metabolic activation. However, no concentration-dependent increases were evident. Under activated conditions, mutagenicity ratios remained below 1.39 at all concentrations (Table 2d). In *E. coli* WP2 *trp uvrA*, CPC produced mutagenicity ratios ranging from 1.18 to 1.67 without metabolic activation and from 1.08 to 1.53 with metabolic activation. These values did not demonstrate a consistent dose–response relationship. For CPC-T3, mutagenicity ratios remained below 1.0 under non-activated conditions. Although ratios exceeding 2.0 were observed at selected concentrations (6.75% and 50%) under metabolic activation, these responses were not dose-dependent (Table 2e).

Revertant colony counts and mutagenicity ratios for CPC and CPC-T3 were compared across all bacterial strains under both non-activated and activated conditions. Under non-activated conditions, a significant increase in revertant colony numbers was observed at 12.5% CPC-T3 in *S. typhimurium* TA100. In contrast, significant decreases were noted at 12.5% and 100% CPC-T3 in *S. typhimurium* TA1537 and at 100% CPC-T3 in *E. coli* WP2 *trp uvrA* when compared with CPC. Under metabolically activated conditions, significant increases in revertant colony numbers were observed at 12.5%, 50%, and 100% CPC-T3 in *S. typhimurium* TA100, as well as at 6.25% CPC-T3 in *E. coli* WP2 *trp uvrA*. Conversely, significant decreases were detected at concentrations ranging from 6.25% to 50% CPC-T3 compared with CPC (Figure 1).

With respect to mutagenicity ratios, a significant increase was detected at 25% CPC-T3 in *S. typhimurium* TA100, whereas significant decreases were observed at 12.5% and 100% in *S. typhimurium* TA1537 and *E. coli* WP2 *trp uvrA* under non-activated condition compared with CPC. Under metabolically activated condition, a significant increase in mutagenicity ratio was detected at 50% CPC-T3 in *S. typhimurium* TA98 relative to CPC (Table 2).

Although statistically significant differences in the revertant colony numbers and mutagenicity ratios were observed at selected concentrations between CPC and CPC-T3 under both metabolic conditions, no concentration-dependent trends were evident. These findings suggested that tocotrienol incorporation did not enhance base-pair substitution or frameshift mutagenic activity. Overall, neither formulation produced reproducible, dose-dependent increases indicative of mutagenicity.

## 4. Discussion

The present study evaluated the mutagenic potential of CPC with and without palm tocotrienol incorporation using the bacterial reverse mutation assay. CPC, with or without palm tocotrienol, did not induce consistent or dose-dependent increases in revertant colony formation across the tested strains, either in the absence or in the presence of metabolic activation. Although isolated increases in mutagenicity ratios were observed in certain strains at individual concentrations, these effects were not reproducible across doses and did not meet the biological criteria for a positive mutagenic response. Therefore, both formulations were considered non-mutagenic under the conditions of this study.

As CPC is intended for biomedical applications involving direct contact with host tissues, the absence of mutagenic activity provides an important initial indication of genetic safety. The biological evaluation of implantable biomaterials was conducted within a tiered framework as outlined in ISO 10993-3 [11]. Within this context, the bacterial reverse mutation assay (also known as the Ames test) is widely used as a first-line screening tool for detecting gene mutations. This assay identifies mutations that restore the function of genes required for histidine synthesis in *S. typhimurium* or tryptophan synthesis in *E. coli* [12]. It is commonly applied in the early phase of safety evaluation due to its sensitivity, reliability, and regulatory acceptance, particularly for identifying potential mutagenic impurities or degradation products that may be released from biomaterials. However, a comprehensive genotoxicity assessment generally requires additional in vitro and in vivo assays. Therefore, the findings of the present study should be interpreted as part of an early-stage biological safety evaluation.

The validity of the assay in this study was supported by negative control values that were comparable to those typically reported for the respective tester strains [13]. This consistency indicated that the bacterial cultures were viable, the agar plates were properly prepared, and incubation conditions were appropriate, thereby minimising the likelihood of contamination or procedural failure [14]. In addition, the robust increases observed in the positive control groups under both non-activated and metabolic activated conditions confirmed the expected responsiveness of the tester strains and demonstrated the functional integrity of the metabolic activation system. These observations are consistent with OECD Test Guideline No. 471 for bacterial reverse mutation test, which specifies that negative controls should yield expected background revertant numbers whereas positive controls should produce marked increases to verify the validity and sensitivity of the test system [15].

According to OECD Test Guideline No. 471, the evaluation of mutagenicity is based on several criteria: (a) the presence of a dose–response relationship; (b) reproducibility across replicates; (c) magnitude and consistency of the increase; and (d) biological relevance relative to historical control ranges [15]. Although isolated elevations in mutagenicity ratio were observed in *S. typhimurium* TA1537 for CPC under non-activated conditions and in *E. coli* WP2 *trp uvrA* for CPC-T3 in the presence of metabolic activation, there were no consistent increases observed across multiple concentrations or bacterial strains. Additionally, these findings were not reproducible across replicates and remained within the expected range of biological variability for the assay. Therefore, these isolated increases were interpreted as sporadic or biologically non-relevant fluctuations attributable to experimental variability rather than evidence of true mutagenic potential.

Metabolic activation is a critical component of the bacterial reverse mutation assay because the bacterial tester strains lack the complex metabolic enzymes present in eukaryotic organisms. Consequently, certain compounds may not be directly mutagenic in their parent form but can acquire mutagenic potential only after enzymatic conversion. Such substances are referred to as pro-mutagens. Following biotransformation, these compounds may be metabolised into reactive electrophilic intermediates capable of damaging deoxyribonucleic acid (DNA) and inducing mutations [16]. To address this limitation, an exogenous metabolic activation system is incorporated into the assay as the S9 fraction, typically prepared from rodent liver homogenates. The S9 mix provides mammalian metabolic enzymes that simulate xenobiotic metabolism and convert pro-mutagens into reactive metabolites [17]. Therefore, conducting tests in the absence and presence of S9 ensures that mutagenic effects arising from either the original compound or its metabolites are adequately detected.

The incorporation of palm tocotrienol into CPC was examined to determine whether its addition could influence the mutagenic safety profile of the cement formulation. Tocotrienols, members of the vitamin E family, are widely recognised for their potent antioxidant, anti-inflammatory, and osteogenic properties [18,19], making them attractive candidates for incorporation into bone-regenerative biomaterials. However, the inclusion of bioactive phytochemicals also necessitates evaluating whether metabolic transformations or degradation products could pose genotoxic concerns.

In the present study, CPC and CPC-T3 did not produce consistent or dose-related increases in revertant colony numbers under either non-activated or metabolic-activated conditions across all tester strains. This indicates that neither formulation exhibited mutagenic activity in bacteria nor generated mutagenic intermediates following metabolic conversion. The comparable responses observed under both metabolic conditions further suggested that metabolic transformation did not alter the genetic safety profile of these materials. Importantly, tocotrienol incorporation did not confer additional mutagenic activity beyond that of CPC alone. Moreover, the expected increases in revertant colonies from the positive controls confirmed that the metabolic activation system was functional and capable of detecting known promutagens. Collectively, these findings support the conclusion that CPC and CPC-T3 are unlikely to pose a mutagenic risk under both direct exposure and metabolically activated conditions.

CPC has been widely reported to exhibit excellent biocompatibility, osteoconductivity, and resorbability. It is commonly used in orthopaedic and dental applications due to their chemical similarity to natural bone [20]. Previous studies have demonstrated that CPC-based formulations do not exhibit genotoxic or mutagenic effects in standard screening assays [21,22], supporting their suitability for clinical use. The absence of consistent increases in revertant colony numbers observed in the present study was in line with earlier reports indicating that calcium phosphate-based biomaterials are not associated with mutagenic risk. This further reinforced the genetic safety of CPC as a scaffold or bone substitute material. In addition, the incorporation of palm tocotrienol did not alter the overall mutagenicity profile, which was consistent with literature suggesting that vitamin E derivatives are typically non-genotoxic [23]. Tocopherols and tocotrienols have been extensively studied for their antioxidant and cytoprotective properties [24,25]. They are generally regarded as biologically safe in toxicological contexts [26,27]. The present findings align with the broader evidence indicating that both calcium phosphate biomaterials and vitamin E-derived bioactives possess low genotoxic potential. These comparisons support the conclusion that CPC and CPC-T3 remain promising candidates for further development in bone regenerative applications without introducing additional mutagenic concerns.

The present study is limited to an initial genotoxicity screening using the Ames test and does not constitute a comprehensive biological safety evaluation. The Ames test is an in vitro bacterial screening assay that does not fully replicate the complexity of mammalian DNA repair mechanisms or in vivo exposure conditions, thereby limiting its ability to accurately predict biological responses under physiological conditions. This study employed extract-based testing, which is widely used in biomaterial safety evaluation to evaluate complex mixtures of leachable components released from the material rather than isolated compounds. Although this approach aligns with established guidelines, it does not capture the complex biological interactions associated with direct material-tissue interactions, including surface-mediated effects and long-term implantation responses. In addition, the assay does not account for prolonged biological responses such as chronic toxicity, degradation-related effects, or sustained interactions with surrounding tissues. Given these limitations, future studies are recommended to incorporate mammalian genotoxicity assays (such as the micronucleus or comet assay) to confirm genetic safety along with independent cytotoxicity assessment using mammalian cell-based system, such as 3-(4,5-dimethylthiazol-2-yl)-2,5-diphenyltetrazolium bromide (MTT), live/dead assays, LDH release, or apoptosis assays to provide complementary insights into cell viability and biocompatibility. In the present study, cytotoxicity was not quantitatively assessed. Therefore, the maximum test concentration was not established based on a maximum tolerated dose. The highest concentration tested was the undiluted extract (100%), which was used to represent the maximum level of leachable substances released under the defined conditions.

Another limitation is the lack of comprehensive structural and chemical characterisation of CPC. Techniques such as phase identification by X-ray diffraction (XRD), compositional analysis by X-ray fluorescence (XRF), evaluation of chemical interactions using Fourier-transform infrared spectroscopy (FTIR), elemental analysis by energy-dispersive X-ray spectroscopy (EDS), microstructural examination via scanning electron microscopy (SEM), and mechanical testing (e.g., compressive strength, bending strength, or fatigue behaviour) were not performed. In addition, parameters such as porosity, pore size distribution, and pore interconnectivity (which are the key determinants of cell infiltration, osseointegration, and fluid transport in bone substitute materials) were not assessed. These further restrict the depth of interpretation regarding the relationship between material properties and the observed biological responses as well as limit the assessment of material’s suitability for load-bearing or structural applications. Future work should therefore include detailed physicochemical, microstructural, and mechanical characterisation to enable more robust structure-function correlations.

The physicochemical properties of the extracts, including pH and the stability of tocotrienol in aqueous conditions, were not evaluated in this study. These factors may influence bacterial viability and the effective exposure of test substances, which should be considered in future investigations. Nevertheless, the acceptable performance of assay controls indicated that the test system remained functionally valid under the experimental conditions. The absence of a tocotrienol-only group also limits the ability to determine whether the observed effects were attributable to the isolated compound or to its interaction with the CPC matrix. Inclusion of a tocotrienol-only group would therefore allow direct evaluation of its intrinsic mutagenic potential and help to distinguish any interaction effects between palm tocotrienol and the CPC matrix. In addition, quantifying the concentration of tocotrienol released from the CPC matrix is important for establishing a clearer relationship between exposure dose and biological response. In the present study, the release profile of tocotrienol was not determined, which limits the ability to directly correlate the absence of mutagenicity with the actual concentration to which the bacterial system was exposed. Inclusion of release kinetics data (e.g., following 24 h incubation and extended time points) would strengthen the interpretation of the findings by providing a more accurate assessment of exposure levels.

Finally, this study was conducted under controlled in vitro conditions, which do not fully reproduce the complexity of the in vivo physiological environment. Factors such as enzymatic activity, pH variation, fluid dynamics, and interactions with biological components (e.g., blood and extracellular matrix) may influence material behaviour and biological responses. Therefore, further studies using more complex biological systems are warranted to strengthen the translational relevance of these findings. It is also important to note that this study utilised the Ames test as an initial screen for mutagenicity. In addition, the biological relevance of the results was considered first, whereas the statistical analysis was used to support the evaluation of the test results in this study. Hence, the statistical significance was not the determining factor for a positive response.

Several key strengths of this study should be highlighted. This work provides a comprehensive evaluation of the mutagenic safety profile of CPC with and without palm tocotrienol incorporation using a standardised bacterial reverse mutation assay. The inclusion of five internationally recommended bacterial strains, including base-pair substitution and frameshift mutation-sensitive systems, ensures broad coverage for detecting different types of gene mutations. In addition, assessments were performed under both non-activated and metabolic-activated conditions, allowing evaluation of direct mutagenicity as well as potential formation of metabolite-mediated mutagenic intermediates. The use of appropriate negative and positive controls further reinforced the validity of the assay as well as confirmed the sensitivity and reliability of the experimental system. Importantly, this study addresses a significant knowledge gap as genotoxicity data on biofunctionalised CPC formulations incorporating tocotrienols remain limited. These findings provide valuable preclinical evidence supporting the genetic safety of CPC-T3 as a promising biomaterial for bone regenerative applications, serving as a foundation for further translational development of tocotrienol-enhanced CPC.

## 5. Conclusions

CPC with and without the incorporation of palm tocotrienol did not exhibit mutagenic activity under the conditions of the bacterial reverse mutation assay. These findings indicate the absence of detectable mutagenic effects in this bacterial system and support its use through an initial genotoxicity screening approach. Nonetheless, these results should be interpreted within the limitations of the assay and do not constitute a comprehensive evaluation of biological safety or suitability for implantable applications. Further evaluations using mammalian genotoxicity assays and in vivo biocompatibility studies are warranted to further characterise the safety profile and to support future translational development.

## Figures and Tables

**Table 1:** The bacterial strains used in the bacterial reverse mutation assay (Ames Test).

Strain	Lot Number	Type of Mutation
*S. typhimurium* TA100	5736D	Base-pair substitution
*S. typhimurium* TA98	5722D	Frameshift mutation
*S. typhimurium* TA1535	70034990	Base-pair substitution
*S. typhimurium* TA1537	70028204	Frameshift mutation
*E. coli* WP2 *trp uvrA*	5894D	Base-pair substitution

**Table 2:** Mutagenicity ratio of CPC and CPC-T3 across bacterial strains in the absence and presence of metabolic activation.

Concentration	CPC (−S9)	CPC-T3 (−S9)	CPC (+S9)	CPC-T3 (+S9)
(a) *S. typhimurium* TA100				
6.25%	0.961 ± 0.209	1.115 ± 0.017	1.097 ± 0.169	1.137 ± 0.156
12.5%	0.912 ± 0.145	1.202 ± 0.128	1.152 ± 0.162	1.223 ± 0.142
25%	0.952 ± 0.121	1.202 ± 0.071 ^#^	1.087 ± 0.086	1.001 ± 0.068
50%	0.950 ± 0.306	1.117 ± 0.150	0.990 ± 0.066	1.056 ± 0.114
100%	1.017 ± 0.160	1.138 ± 0.085	1.031 ± 0.050	1.071 ± 0.082
(b) *S. typhimurium* TA98				
6.25%	1.685 ± 0.187	1.346 ± 0.468	1.018 ± 0.496	0.753 ± 0.301
12.5%	1.852 ± 0.257	1.832 ± 0.358	1.000 ± 0.377	0.714 ± 0.177
25%	1.374 ± 0.374	1.181 ± 0.188	1.073 ± 0.574	0.788 ± 0.153
50%	1.496 ± 0.563	1.408 ± 0.244	1.293 ± 0.268	0.585 ± 0.163 ^#^
100%	1.263 ± 0.328 ^b^	1.480 ± 0.198	1.192 ± 0.373	0.730 ± 0.292
(c) *S. typhimurium* TA1535				
6.25%	1.267 ± 0.356	2.319 ± 0.898	1.040 ± 0.658	1.085 ± 0.174
12.5%	1.623 ± 0.544	1.542 ± 0.686	1.321 ± 0.158	1.026 ± 0.266
25%	1.429 ± 0.515	1.194 ± 0.173	1.242 ± 0.095	1.344 ± 0.207 ^b^
50%	1.389 ± 0.347	2.028 ± 0.555	1.413 ± 1.107	1.270 ± 0.110
100%	1.097 ± 0.340	1.653 ± 0.618	1.253 ± 0.032	1.122 ± 0.113
(d) *S. typhimurium* TA1537				
6.25%	1.491 ± 0.317	1.375 ± 0.331	1.365 ± 0.410	1.241 ± 0.399
12.5%	2.018 ± 0.259	1.208 ± 0.144 ^#^	1.332 ± 0.317	1.206 ± 0.258
25%	1.271 ± 0.302 ^b^	1.042 ± 0.072	1.340 ± 0.500	1.179 ± 0.256
50%	1.194 ± 0.255 ^b^	1.208 ± 0.473	1.100 ± 0.325	1.213 ± 0.468
100%	1.791 ± 0.314	1.000 ± 0.331 ^#^	1.078 ± 0.360	1.385 ± 0.914
(e) *E. coli* WP2 *trp uvrA*				
6.25%	1.433 ± 0.208	0.917 ± 0.382	1.179 ± 0.115	2.174 ± 1.184
12.5%	1.189 ± 0.019	0.857 ± 0.143 ^#^	1.184 ± 0.094	1.689 ± 0.800 ^a^
25%	1.344 ± 0.486	0.714 ± 0.286	1.081 ± 0.073	1.567 ± 0.330
50%	1.533 ± 0.611	0.851 ± 0.248	1.534 ± 0.218	2.056 ± 1.084
100%	1.667 ± 0.208	0.393 ± 0.129 ^#^	1.213 ± 0.169	1.793 ± 0.200

Data are presented as mean ± standard deviation. Symbol ‘^#^’ indicates significant difference as compared to CPC at the same concentration; ‘^a^’ indicates significant difference as compared to 6.25%; ‘^b^’ indicates significant difference as compared to 12.5%.

## Data Availability

The original contributions presented in this study are included in the article. Further inquiries can be directed to the corresponding author.

## Abbreviations

The following abbreviations are used in this manuscript:

ANOVA	Analysis of variance
CPC	Calcium phosphate cement
CPC-T3	Tocotrienol-enriched calcium phosphate cement
DMSO	Dimethyl sulfoxide
DNA	Deoxyribonucleic acid
MCPM	Monocalcium phosphate monohydrate
OECD	Organisation for Economic Co-operation and Development
OPG	Osteoprotegerin
RANK	Receptor activator of nuclear factor kappa-B
RANKL	Receptor activator of nuclear factor kappa-B ligand
β-TCP	Beta-tricalcium phosphate
